# Changes in the gut microbiome and metabolome in a rat model of pulmonary arterial hypertension

**DOI:** 10.1080/21655979.2021.1952365

**Published:** 2021-08-18

**Authors:** Wei Hong, Qiudi Mo, Luyao Wang, Fang Peng, Yuming Zhou, Weifeng Zou, Ruiting Sun, Chunxiao Liang, Mengning Zheng, Haiqing Li, Dongxing Zhao, Mi Gao, Jinding Pu, Bing Li, Pixin Ran, Gongyong Peng

**Affiliations:** a State Key Laboratory of Respiratory Disease, National Clinical Research Center for Respiratory Disease, National Center for Respiratory Medicine, Guangzhou Institute of Respiratory Health, The First Affiliated Hospital of Guangzhou Medical University Guangzhou, Guangzhou, Guangdong, China; b GMU-GIBH Joint School of Life Sciences, Guangzhou Medical University, Guangzhou, Guangdong, China; c Department of Respiratory, The Second Affiliated Hospital of Guangzhou Medical University, Guangzhou, Guangdong, China; d Department of Critical Care Medicine, The Third Affiliated Hospital of Guangzhou Medical University Guangzhou, Guangzhou, Guangdong, China; e State Key Laboratory of Respiratory Disease, Guangzhou Chest Hospital, Guangzhou, Guangdong, China; f Department of Respiratory and Critical Care Medicine, Guizhou Provincial People’s Hospital, Guiyang, Guizhou, China; g Department of Respiratory Medicine, The Third Affiliated Hospital of Guangzhou Medical University, Guangzhou, Guangdong, China

**Keywords:** Pulmonary arterial hypertension, gut, microbiome, metabolome

## Abstract

The gut microbiota is widely considered to be involved in several diseases, including atherosclerosis, obesity, chronic obstructive pulmonary disease (COPD) and pulmonary arterial hypertension (PAH). This study aimed to determine if changes in the gut microbiome and metabolome play a major role in the early pathogenesis of PAH. Male Wistar rats were injected with monocrotaline (MCT) (55 mg/kg) at day 1 and injected with calcium-sensing receptor (CaSR) antagonist NPS2143 (4.5 mg/kg/d) from days 1 to 21. Fecal samples were obtained. The gut microbiota and metabolome were analyzed by 16S rRNA gene sequencing and mass spectrometry-based analysis to investigate the effect of PAH in this rat model. MCT injection had a marked effect on the composition of the gut microbiota. This finding was further confirmed by metabolomic analysis with identification of several metabolites relevant to the gut microflora. However, NPS2143 partially abrogated this intestinal flora disorder and reversed fecal metabolite abnormalities. In conclusion, our study shows correlations between changes in the gut microbiome and the metabolome in PAH, which are affected by NPS2143.

## Graphical abstract

**Figure:**
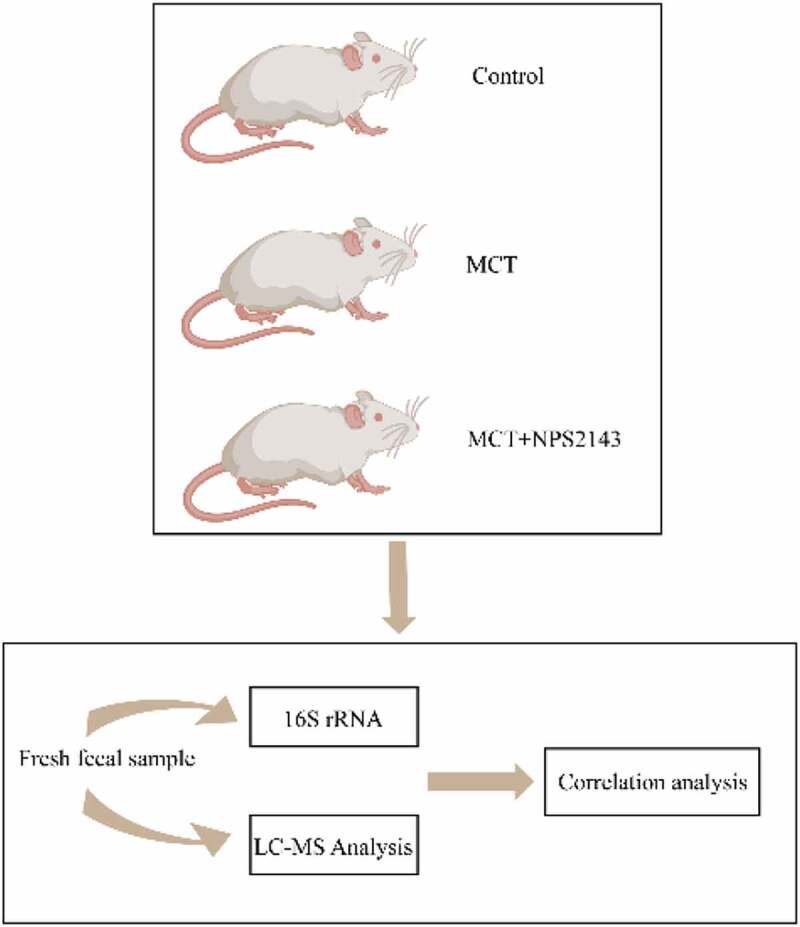

## Introduction

Pulmonary arterial hypertension (PAH) has the distinctive features of decreased pulmonary arterial compliance (PAC) together with elevated pulmonary vascular resistance (PVR). Therefore, PAH could become fatal and is thus considered to be a major concern in human health [1]. Various issues with PAH include a lack of rapid and reliable screening and detection assays for early diagnosis of PAH, a lack of specific and reliable biomarkers for PAH, and no novel treatment and therapy are available [2]. Consequently, determination of the mechanism of PAH and development of novel therapeutic treatments are important.

Calcium (Ca^2+^)-sensing receptor (CaSR) is a member of family C of G-protein coupled receptor amino acids. In combination with G protein, multiple intracellular signaling pathways are activated or inhibited [3]. The ligands or activators of CaSR consist of polyvalent cations (e.g., Ca^2+^, Mg^2+^, and Gd^3+^), polypeptides (e.g., amyloid-β peptide), polyamines (e.g., spermine, spermidine, and putrescine), aminoglycoside antibiotics (e.g., neomycin and kanamycin), and amino acids (e.g., phenylalanine, tyrosine, tryptophan, and glutamate). Moreover, synthetic CaSR agonists (calcimimetics) (e.g., NPS-R-568 and NPS-R-467) and CaSR antagonists (calcilytics) (e.g., NPS 2143) affect CaSR function [4]. The main function of CaSR is to maintain the balance of serum Ca^2+^, and it is also involved in cellular proliferation and differentiation, and hormone secretion. CaSR is upregulated with a rise in cytosolic-free Ca^2+^ concentrations triggered by extracellular Ca^2+^ in pulmonary arterial smooth muscle cells in patients with idiopathic pulmonary arterial hypertension. Blocking the pharmacological effect of CaSR considerably alleviates the development and progression of PAH in rats [5].

For the past decade, the human microbiome has been well established a leading factor in human health and is involved in various diseases. Of these, the gut microbiota is considered to be the most important aspect, and it is associated with respiratory diseases (e.g., PAH) through effects on the gut lung axis [6–8]. Studies have also shown an association between the gut microbiota and produced metabolites, which greatly affects PAH and other diseases [9–11]. Callejo et al. [12] reported that a misbalanced bacterial ecosystem might play a pathophysiological role in PAH by altering immunological, hormonal, and metabolic homeostasis. Another study showed that antibiotic-induced modification of the gut microbiota suppressed the development of PAH [13]. Kim et al. [14] reported that PAH-associated species, such as *Blautia, Bifidobacterium*, and *Collinsella*, contributed genes for carnitine metabolism or biosynthesis of arginine, ornithine, and proline. This provided evidence that a change in gut microbiome function in a PAH cohort is closely associated with taxonomic alterations. Patients with PAH have a unique microbiome profile that has a high predictive potential for PAH. Identification and characterization of PAH-specific bacteria and metabolites in the gut could lead to the development of innovative strategies for the control and treatment of PAH.

Multiple omics and bioinformatics analyses are widely used to investigate the molecular mechanism of various diseases [15,16]. However, the combination of determining the gut microbiota and metabolomics analysis in PAH has not been reported. Therefore, in the current study, we investigated the gut microbiota and performed metabolomic analysis to determine the mechanism of how the host and microbiome are involved in PAH in a rat model.

## Materials and methods

### Animal model for PAH

For the animal model, male Wistar rats that weighed from 250 to 350 g and were aged from 6 to 8 weeks old, were used in this study. The acquisition and care of rats are described in the Acknowledgment section. Twenty-four rats, in three groups (control, PAH, and PAH+NPS2143), were used in this study. All experiments on rats were performed by following a standard procedure as previously published. Induction of PAH was performed by monocrotaline (MCT) injection on the first day at a dose of 55 mg/kg. Subsequently, during days 1 to 21, intraperitoneal injection of NPS2143 was performed on a daily basis at a dose of 4.5 mg/kg/day. Finally (day 21), sodium pentobarbital at a dose of 30 mg/kg was used to sacrifice the rats. The development of the PAH model was evaluated by measuring the ratio of the right ventricle/left ventricle + septum, and right ventricular systolic pressure.

### Analysis of the gut microbiota

For analysis of the gut microbiota, we collected and processed fresh fecal samples of rats according to a standard procedure. Template DNA isolation was further performed, followed by sequencing on the Illumina HiSeq 2500 platform. The primer sequences were as follows: 341 F 5′-CCTAYGGGRBGCASCAG-3′ and 806 R 5′-GGACTACNNGGGTATCTAAT-3′. Sequence assembly, quality control, and clustering were then performed.

### Metabolomic profiling by liquid chromatography and mass spectrometry (LC-MS)

Metabolomic analysis of all samples was performed using the Thermo Ultimate 3000 system according to previous studies [17]. Additionally, ESI-MSn analysis was conducted as previously described [18,19].

### Correlation analysis of gut microbiome and host metabolome

The correlation between the gut microbiota and the host metabolome was analyzed. Analysis was performed as previously described [20].

### Statistical analysis

Data are showed as the mean ± standard error. Data were analyzed using one-way analysis of variance. Pearson’s correlation analysis was used to determine the correlation between the gut microbiota and the host metabolome. Relative indices were analyzed using SPSS version 22.0 software. The data were graphically plotted using GraphPad Prism 7. Differences were considered statistically significant at p < 0.05.

## Results

### The gut microbiome is altered in rats with PAH

Ca^2+^ can be a stimulation factor depending on different concentrations, and the receptor for Ca^2+^also plays an important role [5]. Because the effect of NPS 2143 and MCT on PAH still requires further clarification, we analyzed the gut microbiota in rats with PAH to determine the pharmacological effects of these compounds on PAH.

**Figure 1.**
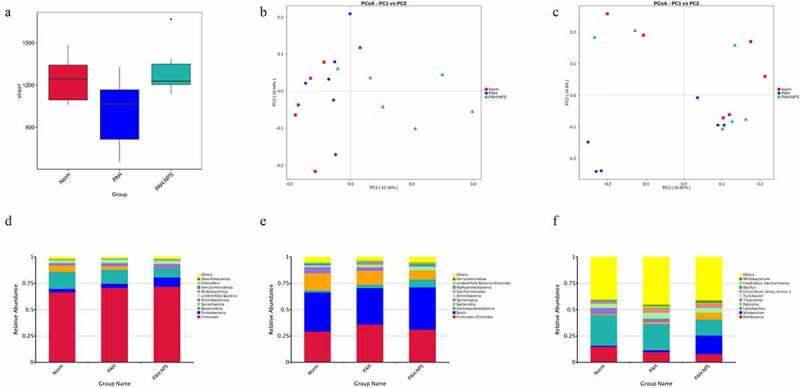
Intestinal microbial diversity and composition among three groups. (a) Variation in diversity within the three groups by Chao index. PCoA plots based on weighted UniFrac metrics (b) and unweighted UniFrac metrics (c). Average relative abundances of dominant bacterial phylum level (d), class level (e), and genus level (f) in the intestine under different treatments

Microbial composition of the gut microbiota showed a lower microbial diversity in the PAH group compared with the control group. However, this change was reversed in the PAH+NPS2143 group, in which NPS2143 led to a significant increase in bacterial diversity (Figure 1(a)). Various bacterial species were identified in all three groups (Figure 1(b) and (c)). Despite sharing similarity in dominating species, changes in the abundance of the gut microbiota were found in all three groups. For the phylum level, the relative abundance of Firmicutes, Proteobacteria, and Actinobacteria was higher in the PAH and PAH+NPS2143 groups than in the control group. However, the relative abundance of Bacteroidota and Spirochaetota was lower in the PAH and PAH+NPS 2143 groups than in the control group (Figure 1(d)). For class level, the relative abundance of Bacilli decreased in the PAH but increased in the PAH +NPS2143 group compared with the control group; Firmicutes-Clostridia increased in the PAH group but decreased in the PAH +NPS2143 group, and Gammaproteobacteria was increase in the PAH and PAH+NPS 2143 groups compared with the control group. However, the relative abundance of Bacteroidia and Spirochaetia was lower in the PAH and PAH+NPS2143 groups than in the control group (Figure 1(e)). For the genus level, the relative abundance of Allobaculum, Ralstonia, and Bifidobacterium was higher in the PAH and PAH+NPS2143 groups than in the control group. However, the relative abundance of Lactobacillus and Romboutsia was lower in the PAH and PAH+NPS2143 groups than in the control group. Additionally, the relative abundance of Turicibacter, Candidatus_Saccharimonas, and Clostridium_sensu_stricto_1 was increased in the PAH group but decreased in the PAH+NPS 2143 groups (figure 1(f)).

**Figure 2.**
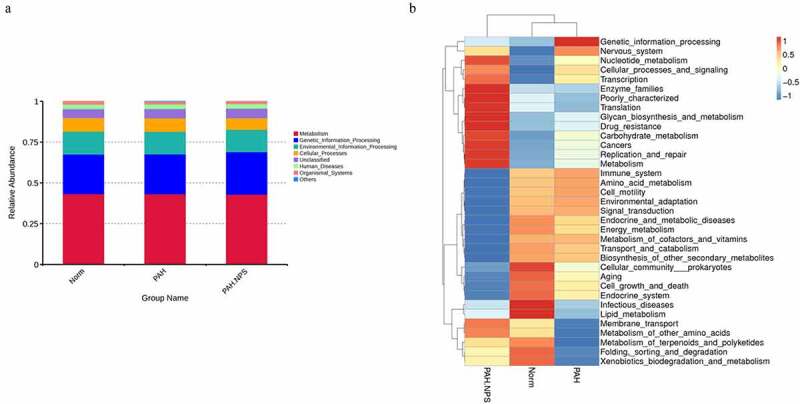
Genes were involved in the pathogenicity of the gut bacteria among three groups. (a) Genes involved in the regulation. (b) All comparisons genes listed in the heatmap

Using the Tax4Fun package, we found that a few pathways were involved in the pathogenicity of the gut bacteria in PAH (Figure 2(a)). These pathways were cellular processes, environmental information processing, genetic information processing, human diseases, metabolism, and organismal systems. Figure 2(b) shows the significantly different functional genes associated with PAH in the three different groups.

### The fecal metabolome is altered in rats with PAH

**Figure 3.**
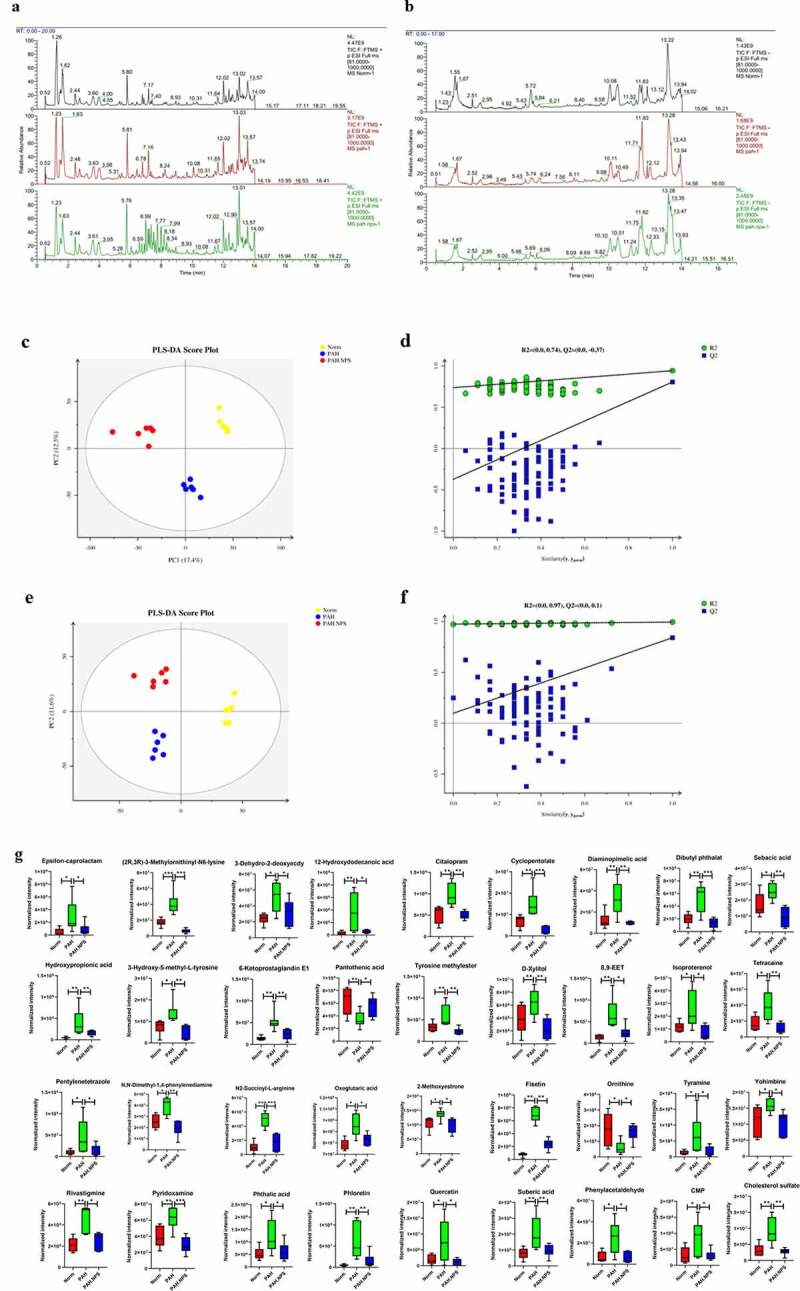
Effect of disruption on the fecal metabolome. Derived PLS-DA score plots and corresponding permutation testing of PLS-DA from the LC-MS metabolite profiles among three groups. Typical LC/MS total ion current (TIC) chromatograms of non target metabolomics from three groups in positive (a) and negative (b) modes. (c) PLS-DA score plot of positive ions. (d) Permutation testing of positive ions. (e) PLS-DA score plot of negative ions. (f) Permutation testing of negative ions. (g) Fecal metabolites disorder among three groups. The data are presented as the mean ± SD, significant differences between groups are indicated as ***P < 0.001, **P < 0.01 and *P < 0.05

In recent years, alterations in the metabolome due to the microbiome have been reported. Accordingly, we investigated the association between the gut microbiota and the metabolome in this study. Typical LC/MS total ion current (TIC) chromatograms in positive and negative modes of nontarget metabolomics from three groups were shown in Figure 3(a-b). Visual inspection of these spectra showed obvious difference among the three groups. Using our optimized LC/MS analysis protocol in association with a software-based peak deconvolution procedure, the most peaks were identified. Regardless of positive or negative ionization modes, a significant difference was found for all three groups (Figure 3(c–f)). As shown in Figure 3(d) and F, R2, and Q2 are similar, indicating that each of the subjects contributes equally and uniformly to the observed

group separation. Hence, the modeling of PLS-DA utilized in this study is stable and reproducible.

This finding indicated that MCT and NPS2143 may be capable of altering metabolomics in rats. A similar finding was also observed (Figure 3(g)). We found significantly dysregulated metabolites in the PAH group compared with the control group. Among these metabolites, the levels of 34 were higher, while those of 2 were lower in the PAH group than in the control group. However, these changes were significantly abrogated in the PAH+NPS 2143 group. Therefore, dramatic changes in metabolites of the gut ecosystem appeared to occur in NPS 2143-treated rats with PAH.

**Figure 4.**
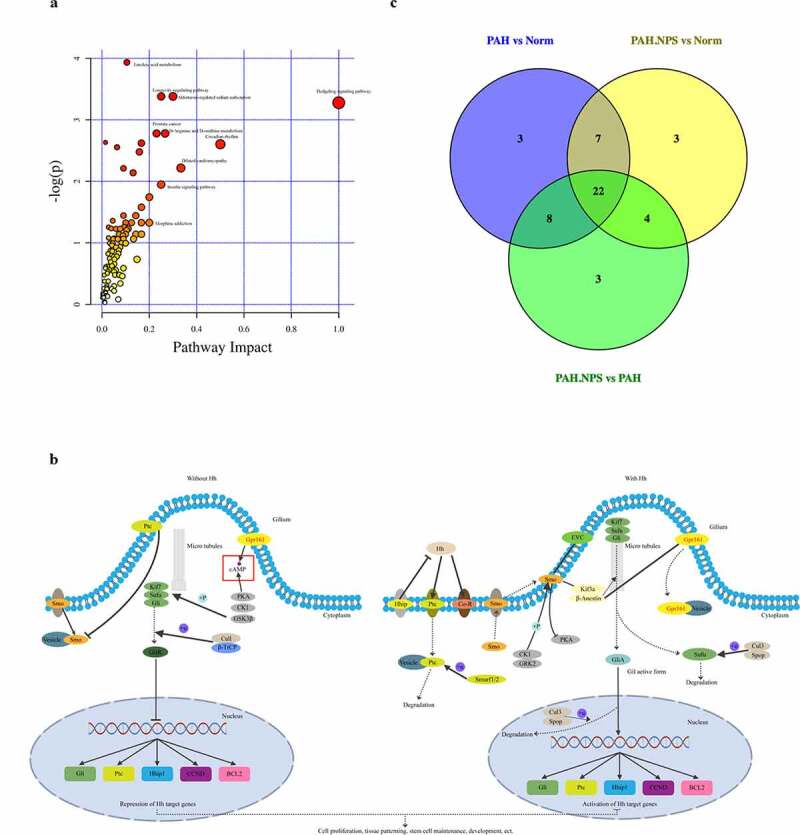
The most enriched KEGG pathways analysis. (a) Ingenuity pathway analysis. The size and color are based on the p-value and impact value, small p-value and big pathway impact value indicate that the pathway is greatly influenced. (b) Overall perspective of hedgehog signaling pathway metabolism map. (c) Venn diagram for common pathway among the three groups

The above-mentioned significantly dysregulated metabolites were enriched in different pathways (Figure 4(a)). On the basis of their p value or impact value, 22 metabolic pathways were identified (Table 1) and KEGG annotation was performed (Figure 4(b)). The Venn diagram of common pathways among the three groups compared with each other is shown in Figure 4(c). Therefore, untargeted metabolomics support the global changes in metabolomics predicted from related changes in the composition of the microbiome.

**Table 1.** Results from Pathway Analysis

Pathways name	Total	Hits	Raw p	-log(p)	Holm adjust	FDR	Impact
Hedgehog signaling pathway	1	1	0.037707	3.2779	1	1	1
Longevity regulating pathway – multiple species	2	1	0.074004	2.6036	1	1	0.5
Circadian rhythm	2	1	0.074004	2.6036	1	1	0.5
Vasopressin-regulated water reabsorption	2	1	0.074004	2.6036	1	1	0.5
Dilated cardiomyopathy	3	1	0.10894	2.2169	1	1	0.33333
Human papillomavirus infection	3	1	0.10894	2.2169	1	1	0.33333
Aldosterone-regulated sodium reabsorption	8	2	0.034028	3.3806	1	1	0.3
D-Arginine and D-onithine metabolism	11	2	0.062099	2.779	1	1	0.26667
Longevity regulating pathway	8	2	0.034028	3.3806	1	1	0.25
Oocyte meiosis	4	1	0.14257	1.9479	1	1	0.25
Insulin signaling pathway	4	1	0.14257	1.9479	1	1	0.25
Progesterone-mediated oocyte maturation	4	1	0.14257	1.9479	1	1	0.25
Growth hormone synthesis, secretion and action	4	1	0.14257	1.9479	1	1	0.25
Leukocyte transendothelial migration	4	1	0.14257	1.9479	1	1	0.25
Prostate cancer	11	2	0.062099	2.779	1	1	0.23077
MAPK signaling pathway	5	1	0.17494	1.7433	1	1	0.2
Rap1 signaling pathway	5	1	0.17494	1.7433	1	1	0.2
Human T-cell leukemia virus 1 infection	5	1	0.17494	1.7433	1	1	0.2
Prion disease	5	1	0.17494	1.7433	1	1	0.2
PPAR signaling pathway	5	1	0.17494	1.7433	1	1	0.2
Chemokine signaling pathway	5	1	0.17494	1.7433	1	1	0.2
Morphine addiction	8	1	0.26496	1.3282	1	1	0.2

The Total is the total number of compounds in the pathway; the Hits is the actually matched number from the 20 species; the p is the original p value calculated from the enrichment analysis; the Holm p is the p value adjusted by Holm–Bonferroni method; the FDR p is the p value adjusted using False Discovery Rate; the Impact is the pathway impact value calculated from pathway topology analysis.

### Correlation between the gut microbiota and the metabolome

**Figure 5.**
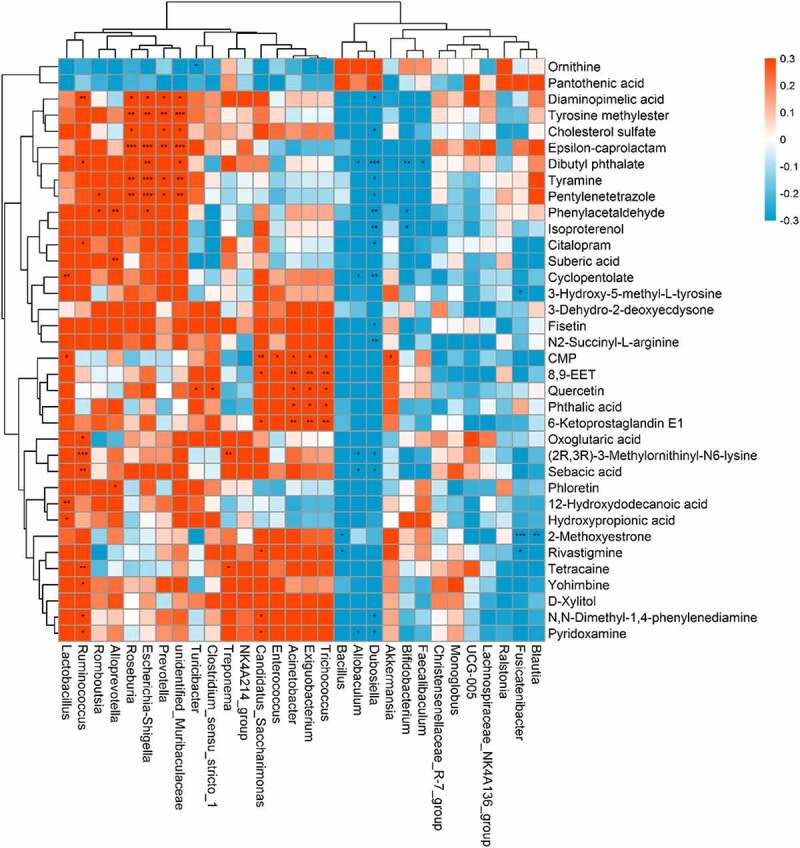
Relationship between gut microbiome and host metabolome. Heat maps indicated positive (red) and negative (blue) correlations between the levels of host metabolites and the identified gut microbiome at the genus levels of NPS2143-treated PAH rats as compared with PAH rats. The legend shows correlation values from −1 to 1 and assigns the appropriate color to them (Red for positive correlations and blue for negative correlations)

The indirect influence of the gut microbiota composition on the host metabolome has been recently reported [21,22]. We further investigated this correlation specifically after NPS 2143 treatment in rats with PAH (Figure 5). Allobaculum was negatively correlated with changes in pyridoxamine, sebacic acid, (2 R,3 R)-3-methylornithinyl-N6-lysine, cyclopentolate, and dibutyl phthalate. Bifidobacterium was negatively correlated with changes in isoproterenol and phenylacetaldehyde. Lactobacillus was positively correlated with changes in hydroxypropionic acid, 12-hydroxydodecanoic acid, CMP, and cyclopentolate. Romboutsia was positively correlated with changes in phenylacetaldehyde and pentylenetetrazole. There were also positive correlations between Candidatus_Saccharimonas and changes in pyridoxamine, N,N-dimethyl-1,4-phenylenediamine, rivastigmine, 6-ketoprostaglandin E1, 8,9-EET, and CMP. These findings suggested correlations between changes in the gut microbiota and the host metabolome, which were attenuated through NPS 2143 treatment in rats with PAH.

## Discussion

There is a considerably large number of species of microorganisms, including bacteria and fungi, in the gastrointestinal tract. Species of microorganisms in the gastrointestinal tract have been widely studied regarding their association with human health [23–25]. The role of gut dysbiosis in the pathogenesis of many diseases is rapidly emerging. In this study, we used multiple omics approaches to study the correlation between the gut microbiota and the host metabolome in rats with PAH and NPS 2143 treatment.

In this study, the gut microbiota was compared between rats with PAH treated with NPS 2143 and control rats. We found a significantly lower α-diversity in the PAH group compared with the control group, which indicated a ‘poor’ health status. However, the α-diversity was restored with NPS 2143 treatment in rats with PAH (Figure 1(a)).

In all three studied groups, we examined the composition of the bacterial gut microbiome in the three groups (Figure 1(b) and (c)). Bacterial composition at the phylum, class, and genus levels was significantly altered by NPS 2143 exposure in rats with PAH. This finding suggests that dysbiosis of the gut microbiota is associated with PAH, which provides new evidence of how the gut microbiome affects the respiratory system. A decrease in potential pathogens and restoration of the normal gut flora by NPS 2143 exposure might alleviate lung injury and improve the host’s metabolism.

In our study, we also showed that changes in the host microbiome reflected alterations in gene expression induced by MCT-treated rats with PAH. MCT exposure also increased the pathogenicity of the gut bacteria in this study (Figure 2(a) and (b)). Genes involved in the regulation of metabolism, genetic information processing, environmental information processing, cellular processes, human diseases, and organismal systems were significantly altered in MCT-treated rats with PAH. NPS 2143 partly reversed this change.

The intestinal microbiome is considered as an additional metabolic organ for the host, and its metabolites can influence the host’s health [26]. Along with changes in the gut microbiome, a disrupted metabolome was also observed in NPS 2143-treated rats with PAH in our study. In the PAH group, 34 and 2 metabolites were up- and downregulated, respectively, compared with the control group (Figure 3(g)). Levels of epsilon-caprolactam, (2 R,3 R)-3-methylornithinyl-N6-lysine, 3-dehydro-2-deoxyecdysone, 12-hydroxydodecanoic acid, citalopram, cyclopentolate, diaminopimelic acid, dibutyl phthalate, suberic acid, hydroxypropionic acid, 3-hydroxy-5-methyl-L-tyrosine, 6-ketoprostaglandin E1, tyrosine methyl ester, d-xylitol, 8,9-EET, isoproterenol, tetracaine, pentylenetetrazole, N,N-dimethyl-1,4-phenylenediamine, N2-succinyl-L-arginine, oxoglutaric acid, 2-methoxyestrone, fisetin, tyramine, yohimbine, rivastigmine, pyridoxamine, phthalic acid, phloretin, quercetin, sebacic acid, phenylacetaldehyde, CMP, and cholesterol sulfate were higher in the PAH group than in the control group. In contrast, pantothenic acid and ornithine levels were significantly lower in the PAH group than in the control group. Changes to these metabolites were significantly abrogated in NPS 2143-treated rats with PAH. A number of previously found important functional metabolites were altered in the fecal samples of MCT-treated rats with PAH, but NPS 2143 abrogated these changes. An increasing number of reports have shown that the gut microbiota is involved in various pathways, including signaling, which is associated with the occurrence and development of PAH. In this study, we provided further insightful analysis on pathways, specifically in consideration of the potential changes and functional roles. We identified 22 pathways (Figure 4(a)). These pathways were the hedgehog signaling pathway, vasopressin-regulated water, the longevity-regulating pathway, longevity regulating pathway-multiple species, dilated cardiomyopathy, circadian rhythm, human papillomavirus infection, aldosterone, D-arginine and D-ornithine metabolism, oocyte meiosis, the insulin signaling pathway, progesterone-mediated oocyte maturation, growth hormone synthesis, leukocyte transendothelial migration, secretion, and action, prostate cancer, the MAPK signaling pathway, prion disease, R human T-cell leukemia virus 1 infection, the chemokine signaling pathway, the peroxisome proliferator-activated receptor signaling pathway, the AP1 signaling pathway, and morphine addiction (Table 1). The hedgehog signaling pathway was the most affected (Figure 4(b)).

In recent years, an increasing number of studies has suggested that the gut microbiota is capable of influencing host metabolism, which eventually leads to changes in the pathogenesis of respiratory diseases [27]. In the current study, the correlation between the microbial composition of the gut microbiota and the metabolome was investigated to determine the mechanisms involved in NPS 2143-treated PAH rats. Importantly, we found the following correlations (Figure 4). The genus Allobaculum was negatively correlated with changes in pyridoxamine, sebacic acid, (2 R,3 R)-3-methylornithinyl-N6-lysine, cyclopentolate, and dibutyl phthalate. Bifidobacterium was negatively correlated with changes in isoproterenol and phenylacetaldehyde. Lactobacillus was positively correlated with changes in hydroxypropionic acid, 12-hydroxydodecanoic acid, CMP, and cyclopentolate Romboutsia was positively correlated with changes in phenylacetaldehyde and pentylenetetrazole. Turicibacter and Clostridium_sensu_stricto_1 were positively correlated with changes in quercetin. Candidatus_Saccharimonas was positively correlated with changes in pyridoxamine, N,N-dimethyl-1,4-phenylenediamine, rivastigmine, 6-ketoprostaglandin E1, 8,9-EET, and CMP. These results indicate that alterations in the microbial composition from the gut microbiota could eventually lead to changes in certain metabolites by NPS 2143 treatment. Additionally, such influence looks more like in a casual way than specifically.

## Conclusion

Our study shows correlations between changes in the gut microbiome and the metabolome in PAH, which are affected by NPS 2143. NPS 2143 treatment attenuates changes in the gut microbiota and subsequent effects on the host metabolome in rats with PAH. The current study may provide important evidence and better understanding of molecular mechanisms, and help in development of novel drug targets and therapeutic treatments for PAH.

## Highlights

The gut microbiome is altered in rats with PAH.

The fecal metabolome is altered in rats with PAH.

Correlation in alteration of gut microbiota and the host metabolome through NPS2143 treatment in PAH rats.
